# *R26R-GR*: A Cre-Activable Dual Fluorescent Protein Reporter Mouse

**DOI:** 10.1371/journal.pone.0046171

**Published:** 2012-09-25

**Authors:** You-Tzung Chen, Ming-Shian Tsai, Tsung-Lin Yang, Amy Tsu Ku, Ke-Han Huang, Cheng-Yen Huang, Fu-Ju Chou, Hsiang-Hsuan Fan, Jin-Bon Hong, Shuo-Ting Yen, Wei-Le Wang, Chang-Ching Lin, Yu-Chen Hsu, Kang-Yi Su, I-Chang Su, Chuan-Wei Jang, Richard R. Behringer, Rebecca Favaro, Silvia K. Nicolis, Chung-Liang Chien, Shu-Wha Lin, I-Shing Yu

**Affiliations:** 1 Graduate Institute of Medical Genomics and Proteomics, National Taiwan University College of Medicine, Taipei, Taiwan; 2 Graduate Institute of Clinical Medicine, National Taiwan University College of Medicine, Taipei, Taiwan; 3 Department of Clinical Laboratory Sciences and Medical Biotechnology, National Taiwan University College of Medicine, Taipei, Taiwan; 4 Department of Otolaryngology, National Taiwan University Hospital and College of Medicine, Taipei, Taiwan; 5 The First Core Laboratory, Branch Office of Medical Research and Development, National Taiwan University College of Medicine, Taipei, Taiwan; 6 Division of Genomic Medicine, NTU Center of Genomic Medicine, National Taiwan University, Taipei, Taiwan; 7 Institute of Statistical Science, Academia Sinica, Taipei, Taiwan; 8 Program in Developmental Biology, Baylor College of Medicine, Houston, Texas, United States of America; 9 Department of Genetics and Center for Stem Cell and Developmental Biology, University of Texas MD Anderson Cancer Center, Houston, Texas, United States of America; 10 Department of Biotechnology and Biosciences, University of Milano-Bicocca, Milano, Italy; 11 Graduate Institute of Anatomy and Cell Biology, National Taiwan University College of Medicine, Taipei, Taiwan; 12 Department of Laboratory Medicine, National Taiwan University Hospital, Taipei, Taiwan; 13 Transgenic Mouse Model Core Facility of the National Research Program for Genomic Medicine, National Taiwan University College of Medicine, Taipei, Taiwan; The Beatson Institute for Cancer Research, United Kingdom

## Abstract

Green fluorescent protein (GFP) and its derivatives are the most widely used molecular reporters for live cell imagining. The development of organelle-specific fusion fluorescent proteins improves the labeling resolution to a higher level. Here we generate a *R26* dual fluorescent protein reporter mouse, activated by Cre-mediated DNA recombination, labeling target cells with a chromatin-specific enhanced green fluorescence protein (EGFP) and a plasma membrane-anchored monomeric cherry fluorescent protein (mCherry). This dual labeling allows the visualization of mitotic events, cell shapes and intracellular vesicle behaviors. We expect this reporter mouse to have a wide application in developmental biology studies, transplantation experiments as well as cancer/stem cell lineage tracing.

## Introduction

Green fluorescent protein (GFP), which was first isolated from jellyfish, is among the most widely used molecular markers in contemporary molecular, cellular and developmental biology [1], [2]. Different from the vital dyes, GFP is a gene product. When the GFP reporter gene is introduced within a transgenic construct or an endogenous locus, its expression pattern reflects the end result of the complex modulating activities of the transcriptional regulatory elements. The GFP gene can also be fused with other gene sequences to generate fusion proteins so that subcellular protein localization and dynamics can be visualized in live cells. For example, the development of organelle-specific fluorescent proteins (FPs) by fusing FPs with other proteins or peptides that target them to different organelles provides a way to follow the dynamic cellular changes in more detail [3]. The development of FP color variants with different excitation or emission wavelengths makes it possible to simultaneously monitor more than one target protein or organelle [4]–[6]. It is also possible to express multiple organelle-FP variants in the same cell [7]–[10]. Combinations of emission colors from FPs create codes to increase labeling diversity for description of complicated systems such as neuronal cell synaptic connections in the brain or the stem cell clonal competitions in the intestine [11], [12].

The *R26* locus was first identified in a gene trapping experiment in mouse embryonic stem cells [13]. There was a ß-galactosidase and neomycin phosphotransferase fusion reporter (*ß-geo*) gene trap construct inserted into this locus that resulted in high level, ubiquitous expression throughout development. Subsequent study revealed that although the insertion of the gene trap cassette disrupted two alternatively spliced transcripts in the gene trap direction, homozygous mutants for this locus were viable with no obvious phenotypic differences from their wild-type littermates [14]. Numerous conditionally activated genes for gain of function studies, reporter cassettes for cell lineage tracing, or suicide genes for genetic cell ablations have been inserted into this locus [15]–[18]. In addition, strategies to accelerate the exploitation of the *R26* locus have been developed [19], [20].

Here we describe the generation of a mouse strain bearing a Cre activable dual fluorescent reporter gene in the *R26* locus. We use a dual fluorescent protein reporter, which encodes for a self-cleavable, bipartite, complex fusion protein that is composed of a chromatin-associated H2B-EGFP fusion protein and a plasma membrane-bound mCherry-GPI (glycosyl-phosphatidyl-inositol signal sequence) fusion protein (*GR*; which stands for green nucleus-red membrane). This dual fluorescent protein reporter allows live imaging of cell cycle status, cell shape changes, and genetically marks cells with a unique appearance under a fluorescent microscope. Cell behaviors during early embryo development and on *in vitro* primary tissue culture of an activated reporter mouse can be consecutively recorded. We expect this dual fluorescent reporter mouse will be a useful tool in developmental biology studies, stem cell and cancer initiating cell lineage tracing as well as transplantation experiments.

## Results

### Generation of the *R26R-GR* Allele in the Mouse

**Figure 1:**
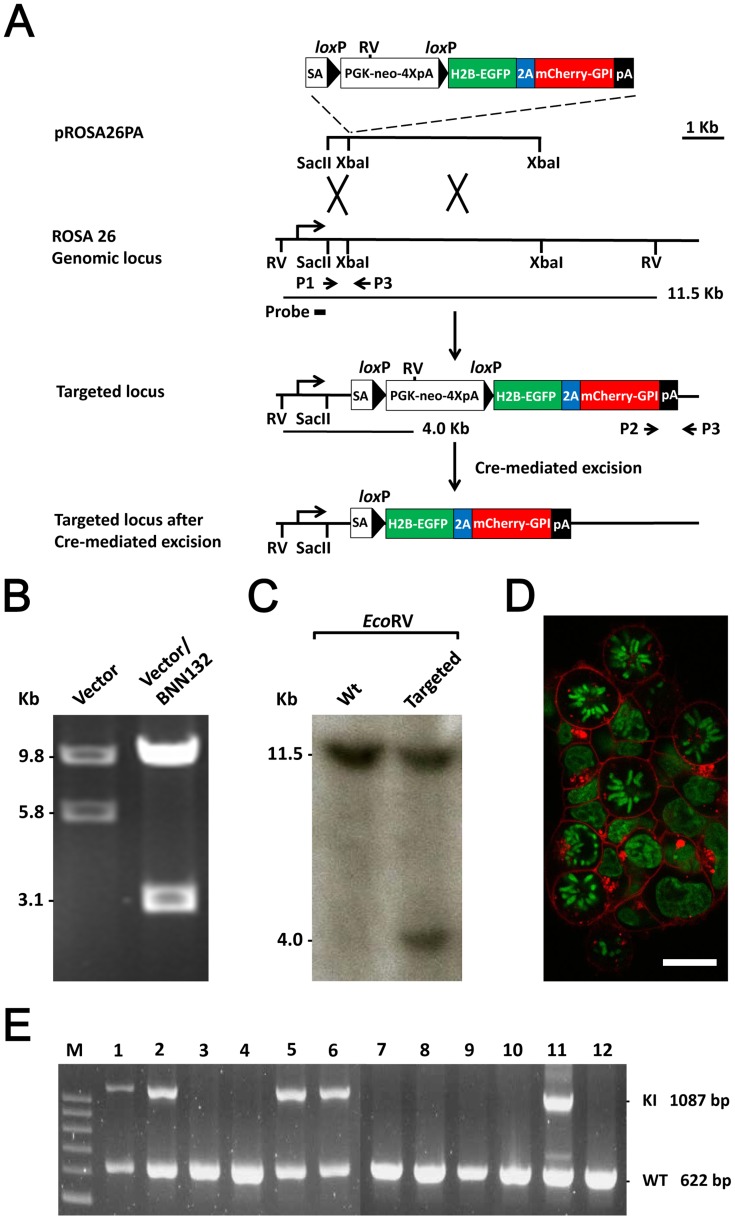
The generation of *R26R-GR* mice. (A) Gene targeting strategy for the Cre activable *R26R-GR* reporter allele. (B) The gene targeting vector contains a *lox*P flanked stop cassette, which can be excised in a Cre-expressing *BNN132 E. coli*, producing a predicted smaller *Pac*I, *Asc*I–restricted DNA fragment. (C) Southern blotting evidence for a *R26* targeted mouse ES cell clone. (D) Transient transfection of a Cre-expressing plasmid, pOG231, activates the dual fluorescent protein reporter in the targeted ES cell clone. Scale bar: 15 µm. (E) Germline transmission demonstrated using a 3-primer PCR-based genotyping strategy. The presence of the wild-type allele results in a 622 bp PCR product amplified by P1 and P3 while the *R26R-GR* targeted allele is amplified by P2 and P3, leading to a 1,087 bp product. Lanes 1, 2, 5, 6, and 11 identify the *R26R-GR* heterozygous mice.

To generate a general reporter mouse, we targeted an inducible dual fluorescent protein reporter cassette (*lox*P-Stop-*lox*P-*H2B-EGFP-2A-mCherry-GPI-p A; R-GR,* which stands for reporter for green-red) to the *R26* locus using a previously described strategy [19] (Figure 1A). The H2B-EGFP encoded a histone 2B protein fused with an enhanced green fluorescent protein which allows the observation of chromatin structure in the nucleus, providing cell cycle information including mitosis [21]. In addtion there was an mCherry-GPI (glycosyl-phosphatidyl-inositol signal sequence) gene encoding a red fluorescent membrane-anchored protein that can highlight cell shape [22]. The two parts of the dual fluorescent protein gene were linked by a sequence encoding the self-cleavage 2A peptide [23]. The 2A peptide allowed efficient dissociation of the two moieties so that the fusion FP variants could localize to different cellular compartments [10], [23].

The targeting vector was constructed and the function of the *lox*P sites flanking the “stop cassette” was tested by transforming the vector into a Cre-expressing *E. coli*., *BNN132* (BD Bioscience Clontech, Mountain View, CA, USA), and the recombined product showed a characteristic *Pac*I and *Asc*I double-restricted fragment length reduction from 5.8 Kb to 3.1 Kb examined by gel electrophoresis. (Figure 1B). Southern blotting using a 5′ external probe was performed to identify gene targeting events (Figure 1C). A long PCR-based approach using a 3′ external genomic primer was used to reconfirm the integrity of the targeted allele (Figure S1). Among 216 G418 resistant ES cell clones screened, 12 targeting events were identified. Three targeted clones were first tested by a transient Cre expression experiment by transfection with a *CMV-Cre* plasmid [7]. All three targeted ES cell clones were competent to express the dual fluorescent label and were used for blastocyst injection to generate germline chimeras (Figure 1D). Germline transmitted pups from chimeras were identified by their coat color from two independent ES cell clones. A 3-primer PCR genotyping strategy was used to identify the presence of the reporter allele (Figure 1E).

### *In vivo* Functional Test of the *R26R-GR* Allele

**Figure 2:**
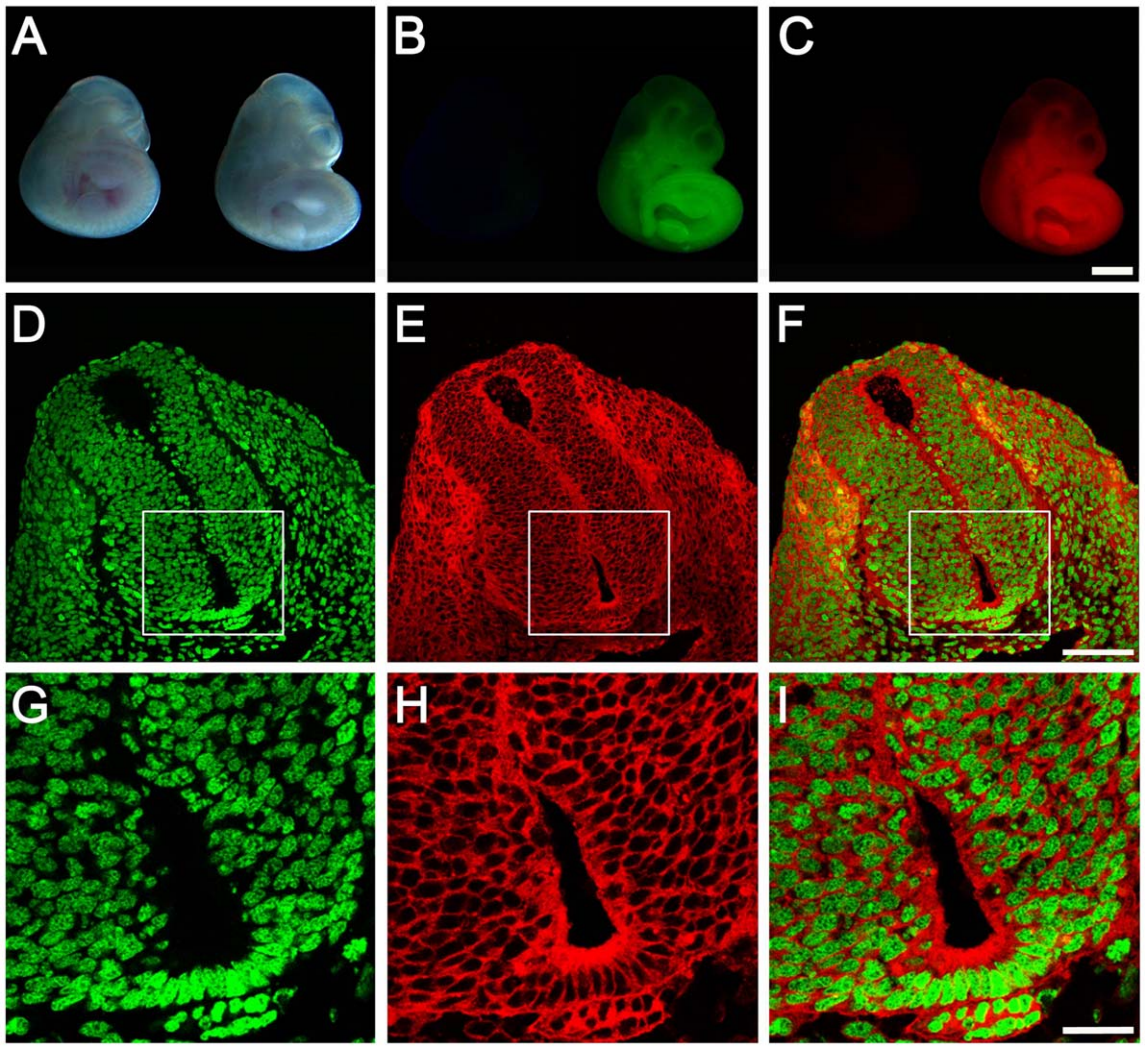
Ubiquitous dual fluorescent protein reporter expression from *R26* resulting from a *Sox2Cre*-mediated activation event. The *R26R-GR* male is crossed with a female *Sox2Cre* transgenic mouse to obtain compound heterozygous progeny with the dual fluorescent protein reporter activated in all tissues of an E10.5 embryo. (A, B, C) Whole mount images of E10.5 heterozygous *R26R-GR* (left) and *Sox2Cre*-activated *R26R-GR* (right) embryos under a fluorescent dissection microscope. The inactivated *R26R-GR* embryo shows neither EGFP nor mCherry signals whereas the compound heterozygous *Sox2Cre/+; R26R-GR/+* embryo emits both green and red lights throughout the body. Scale bar: 1 mm. (D, E, F) Cross section of a E10.5 *Sox2Cre/+; R26R-GR/+* embryo reveals ubiquitous expression of a nucleus localized EGFP and a cell membrane bound mCherry. Scale bar: 75 µm. (G, H, I) Higher magnifications from D–F pictures are shown. Scale bar: 25 µm.

To test whether the targeted *R26R-GR* allele can potentially mark all tissues in the body, a male *R26R-GR* heterozygte was crossed with a female *Sox2Cre* transgenic mouse [24]. *Sox2Cre* is capable of mediating efficient *lox*P recombination in the epiblast before E6.5. Therefore, tissues derived from all three germ layers should be labeled with the dual fluorescent reporter. Compound heterozygous E10.5 embryos carrying a *Sox2Cre* transgenic allele and an *R26R-GR* allele emit both green and red fluorescence under a fluorescent dissection microscope (Figure 2, A–C). The *lox*P site flanking region of the activated *R26-GR* allele was PCR amplified from genomic DNA of these dual fluorescent embryos. PCR product sequencing analysis confirmed that the Cre-mediated recombination excised the “stop” cassette and brought the *GR* reporter gene to a position directly downstream of the *R26* promoter (Figure S2). Total protein extracted from these bigenic embryos was used for Western analysis. Antibodies against GFP and mCherry revealed bands of molecular weight close to 47 KD and 30 KD respectively, indicating an efficient dissociation of the H2B-EGFP and the mCherry-GPI moieties achieved by the self-cleavable 2A peptide (Figure S3). Confocal microscopy of frozen sections revealed the signature red membrane and green nucleus pattern in the skin, neural tube, notochord and other mesenchymal tissues (Figure 2, D–I and Figure S4). This pattern was observed in all of the tissues examined at this developmental stage (data not shown). In contrast, although the dual fluorescent reporter signal was observed in all the major organs in an adult mouse, the subcellular distributions of the mCherry signal displayed cell type specific patterns in different tissues (Figure S5 and Figure S6). The H2B-EGFP patterns also indicated which cells were in the mitotic phase of the cell cycle (Figure 1D, and Figure S7) [21].

### Tissue-specific Activation of the R26R-GR Allele in the Mouse

**Figure 3:**
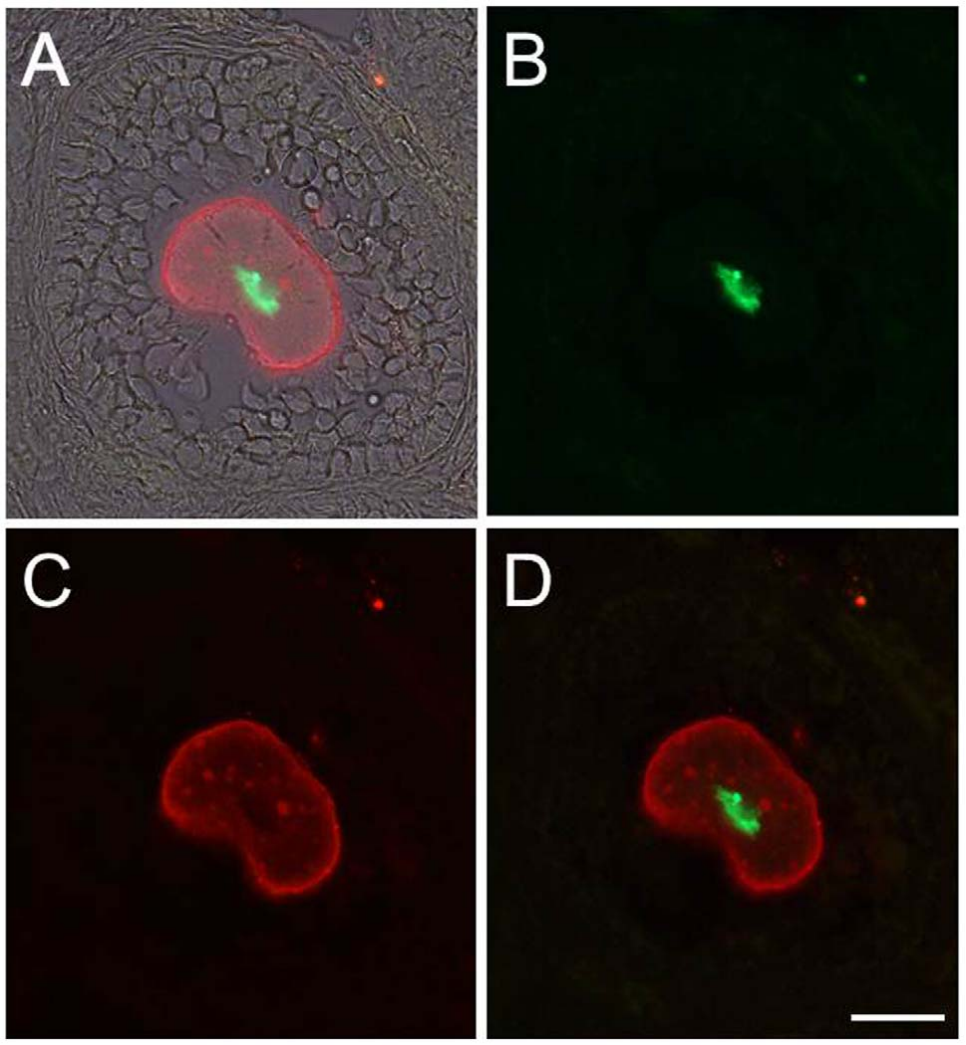
Oocyte-specific dual fluorescent reporter labeling induced by a *Zp3Cre* transgene. Frozen ovary section from an eight week old *Zp3Cre/+; R26R-GR/+* female mouse illustrates a tissue-specific, Cre activable expression of the dual fluorescent reporter gene (*GR*). In the picture, the oocyte is the only cell in the follicle that shows both nuclear EGFP and cell membrane mCherry signals (D). Although the granulosa cells encompassing the oocyte and theta cells surrounding the follicle are observed in the DIC mode, EGFP channel, and mCherry channel overlaid image (A), they emit no fluorescent signals and they are absent in photos taken under EGFP channel (B) and mCherry channel (C). Scale bar: 20 µm.

To test whether the *R26R-GR* allele is responsive to a tissue specific Cre-mediated activation at a later developmental stage, *R26R-GR* homozygous female mice were mated to a *Zp3Cre* transgenic male. *Zp3Cre* was demonstrated to have a growing oocyte-specific Cre expression pattern with a very high Cre-mediated target gene deletion in 100% of oocytes in mature female mice [25], [26]. Ovary cryosections from a resulting 8-week-old *R26R-GR/+; Zp3Cre/+* female revealed an oocyte-specific activation of the dual fluorescent reporter gene (Figure 3). In female mammals, the oogenesis is characterized by a long period of meiotic arrest and lacks the assistance of centrosomes. Each oogonium arrests at the diplotene stage of meiosis I and lies dormant within the follicle which is composed of a protective layer of granulosa cells. During this dictyotene stage, the synaptonemal complex degrades and the homologous chromosomes separate and decondense a bit. However they remain tightly bound at chiasmata until they are separated during anaphase I. In our experiment, the observation of the condensed H2B-EGFP signals in all the oocyte examined is consistent with these characteristics. In addition, we also mated the *R26R-GR* homozygous females with a *Sox2-CreERT2* transgenic male and a *K15-CrePR* male [27], [28]. Compound heterozygous progeny from each cross were used for a tamoxifen or a RU486 induction, respectively. Subsets of *Sox2*-expressing neural progenitor cells in the hippocampus and K15 positive skin stem cells located within the hair follicle of back skin were found to be labeled with the GR-reporter (Figure S8). Thus, the *R26R-GR* allele is responsive to a tissue-specific Cre induction.

### Live Imaging of the Activated *R26-GR* Reporter in Preimplantation Embryos

**Figure 4:**
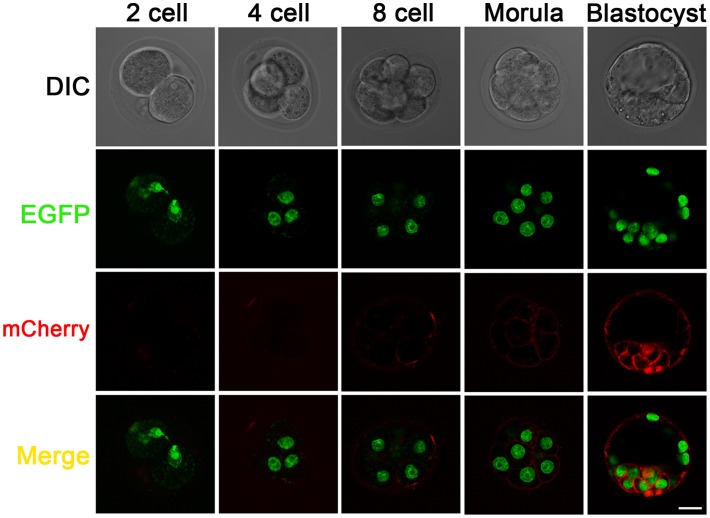
Live images of homozygous *R26-GR* pre-implantation embryos at 2-cell, 4-cell, 8-cell, morula and blastocyst stages. Scale bar: 25 µm. A 3D reconstructed movie taken on an E3.5 blastocyst is provided in the Movie S1.

The *R26* locus was previously reported to be ubiquitously expressed in all tissues throughout development. The activated *R26-GR* allele was tested to determine if there was sufficient expression to allow high-resolution live recording of cellular changes. Rather than the heterozygous, conditionally activated mid-gestation embryos (Figure 2) or mice after birth (Figure 3), we imaged preimplantation embryos homozygous for the activated *R26-GR* allele in an *in vitro* culture system. Images were taken throughout a three day *in vitro* culture period, beginning from a 2-cell stage embryo. Confocal images were taken at 2-cell, 4-cell, 8-cell, morula, and blastocyst stages (Figure 4). No obvious, deleterious effect on embryonic development due to repeated imaging was observed. The result of a scan of a blastocyst at the end of the experiment and 3-D reconstruction of different focal planes is presented as a movie in Movie S1. In the *R26-GR* homozygous embryos we found that the nuclear EGFP signal is intense enough for imaging at all stages examined, however, the membrane mCherry signal was weak from the 2-cell to morula stages. At the blastocyst stage, although a membrane bound mCherry signal became strong, the cytosolic distribution of mCherry also became apparent.

### Time-lapse Imaging of Cell Behaviors in the Heterozygous *R26-GR* Mouse Embryonic Fibroblast Primary Culture

**Figure 5:**
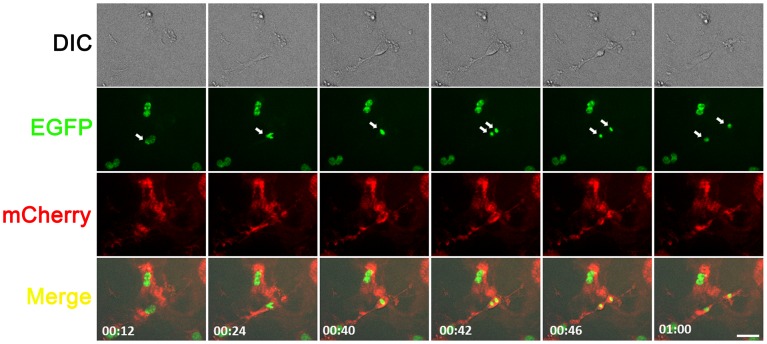
Time–lapse imaging in an E14.5 heterozygous *R26-GR* mouse embryonic fibroblast primary culture. Snapshots from a time-lapse imaging experiment (Movie S2). The elapsed times are given at the lower-left corner in each merged image. A cell division event is recorded in this experiment. Arrowheads indicate a dividing nucleus and her daughters. Scale bar: 50 µm.

To test whether the *R26R-GR* allele provides enough fluorescent signals for recording cell behaviors over time, we performed multi-channel time-lapse recording on a mouse embryonic fibroblast (MEF) primary cultures derived from an E14.5 heterozygous *R26-GR* embryo and successfully captured cell shape changes, movements and mitosis. Snapshots of the time-lapse movie (Movie S2) are presented in Figure 5. These results demonstrated that a single *R26-GR* allele can provide sufficient dual fluorescent protein reporter expression for time-lapse imaging of primary cell cultures.

### mCherry-GPI Labels Endoplasmic Reticulum and Endosome

**Figure 6:**
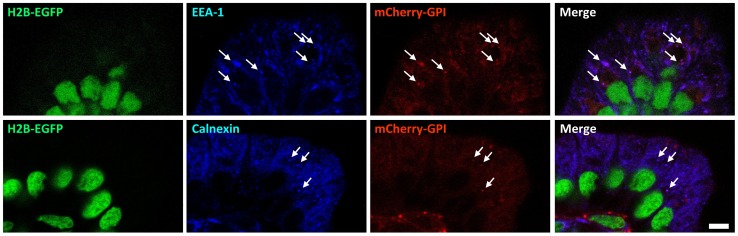
Endoplasmic reticulum (ER) and endosomes labeled by mCherry-GPI in the intestinal epithelia of a P21 heterozygous *R26-GR* pup. Immunohistochemistry using antibodies against a endosome-specific marker (EEA-1)(top row) and an ER-specific marker (calnexin) (bottom row) provides signals coincided with mCherry-GPI distributions (arrows). Scale bar: 5 µm.

**Figure 7:**
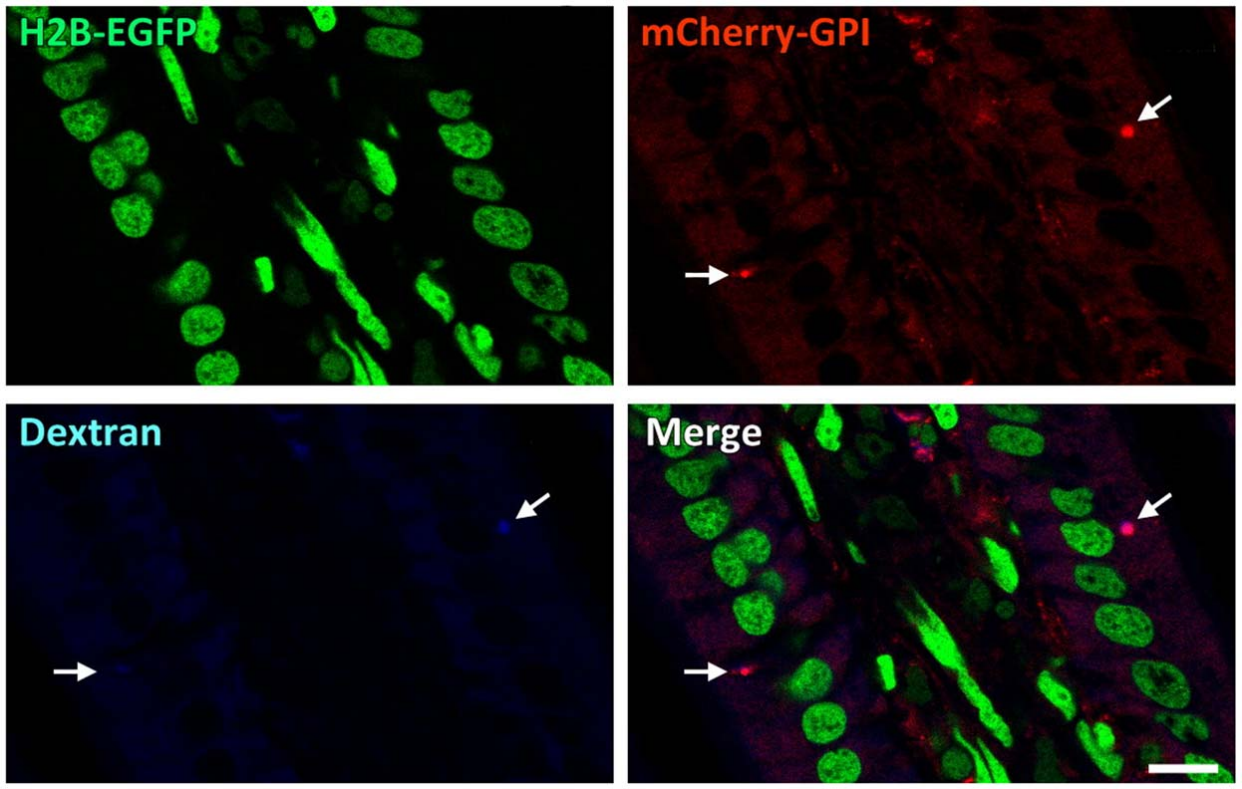
Endocytic events colocalize with mCherry-GPI marked vesicles. Cascade blue-labeled 10-kD dextran is found in mCherry-GPI marked vesicles in the intestinal epithelia of a P21 *R26-GR* heterozygote two hours after gavage feeding (arrows). Scale bar: 10 µm.

Glycosyl-phosphatidylinositol-anchored proteins (GPI-APs) are known to be involved in a diverse range of physiological functions [29]–[34]. As it was mentioned above, there was variation in the subcellular distribution of mCherry in adult, depending upon cell type (Figure 6). In intestinal epithelia, cytosolic red signal was observed but plasma membrane mCherry signal was observed to be patchy. Intense red emissions from vesicles of various sizes were observed (Supplemental Material 6). Immunohistochemistry studies colocalized some of these red vesicles with calnexin and EEA1, markers for endoplasmic reticulum (ER) and endosomes, indicating that at least some of the mCherry vesicles observed are involved in the protein sorting machinery or participated in the endocytic pathway (Figure 6). Frozen intestinal sections of a 10-kD dextran conjugated-dye fed P21 pup also revealed that at least some of the mCherry-GPI labeled vesicles co-localized with the endocytosed cyan signals (Figure 7). These observations were consistent with similar studies reported previously [10], [22].

## Discussion

With the increasing attention focused on stem cell and cancer initiation cell researches, *in vivo* cell-cell interactions and detailed developmental cell lineage relationships are waiting to be revealed [35]. Interdisciplinary research combining transgenic vital labeling techniques and advanced microscopy that allow *in vivo* live imaging at a subcellular resolution are becoming more and more important. The *R26* locus allows ubiquitous, high level gene expression and it is widely used for the generation of reporter mice [13], [15], [16], [19]. Recently organelle-specific fluorescent protein fusions have provided a new way to directly observe cellular and sub-cellular organelle behaviors [36], [37]. Multi-color labeling cassettes mark single cells with different colors or different color combinations on the same cell enable unambiguous assignments of individual cells in a complicated system [11], [12], [38], [39].

In our studies, we used a 2A linked H2B-EGFP and mCherry-GPI reporter cassette which marks the chromatin with green fluorescence and membrane structures with a red fluorescent signal. The self-cleaving 2A peptide uncoupled chromatin-associated EGFP with the membranous inositol-associated mCherry so that cellular activities in different subcellular compartments can be recorded simultaneously using different channels [8]–[10], [40], [41]. We found the green chromatin signal distinguished cell cycle stages in ES cells, *in vitro* cultured MEFs and adult oogonia consistent with previous studies [21], [36], [42]. Histone-fused fluorescent proteins have been found to be useful for computer annotation of live-imaging of the dynamic chromatin structures during cell cycle phases [43], [44]. Time-lapse live-cell image-based high throughput drug screening by directly scoring cellular dynamics therefore can be developed. Our *R26R-GR* mice may serve as primary tissue sources for such screens in the future.

Although mCherry was supposed to be expressed at the same level as EGFP, we consistently observed a lower mCherry signal. This was at least partly due to different physical properties, such as quantum yields, brightness and photostability of the fluorescent proteins. The EGFP was brighter than mCherry by its nature. Also, the subcellular distribution of the mCherry-GPI signal appeared to be affected by GPI anchor addition and/or removal, and/or the GPI protein sorting system that reflect cell physiological conditions. The mCherry-GPI moiety is composed of a pro-acrosin signal peptide fused mCherry and the 5′ end of the mouse Thy-1 GPI-anchoring sequence which should label the plasma membrane outer leaflet [22], [45]–[48]. The mCherry signal we observed is largely consistent with the previously described GPI-anchored fluorescent proteins [22], [45]. Although we observed an evenly labeled plasma membrane in ES cells and oogonia, between the 2-cell to 8-cell stages, we detected very low mCherry signal on the cell membrane. The plasma membrane mCherry signal, as well as some cytosolic mCherry signal, increased siganificantly by the morula stage and became prominent in blastocycsts. Consistent red membrane signal was observed in the post-implantation embryo however a polarized subcellular distribution was observed in cells within the floor plate of the neural tube at E10.5. Elevated mCherry fluorescence was observed in the notochord, neural tube floor plate, sclerotome, the ectoderm-mesenchyme boundary and some limb mesenchyme. Whether these intense mCherry signals reflect certain cell physiological activities is yet to be determined.

In summary, we constructed a potentially very useful dual fluorescent protein reporter mouse line which could be widely applied to a variety of research disciplines. The quantity and subcellular distributions of GPI-anchored mCherry reporter protein in different tissues of an activated reporter mouse may provide insights for GPI protein- regulated developmental and physiological events.

## Materials and Methods

### Targeting Vector Construction

The H2B-EGFP-2A-mCherry-GPI-pA (GR-pA) cassette was retrieved from pXL-T3-Neo-UGm-cHS4X [42]. The cassette was then introduced between the *Xho*I and *Not*I sites of pBigT, which contains a *lox*P-flanked PGK-neo stop cassette upstream of the cloning sites [19]. To generate the *R26R-GR* targeting construct for Cre-mediated conditional expression of organelle-specific bi-fluorescence, the derived pBigT-GR-pA was doubly restricted with *Pac*I and *Asc*I to excise a fragment, including the *lox*P-flanked stop cassette and GR-pA, for subcloning into pROSA26PA [19] with 5′- and 3′- homologous arms to target the *R26* locus. The final construct was verified by DNA sequencing and linearized with *Kpn*I, followed by ethanol precipitation for gene targeting.

### ES Cell Gene Targeting and Reporter Mouse Generation

To generate *R26R-GR* mice, the targeting vector was transfected into R1 ES cells (129X1/SvJ_129S1) by electroporation [49]. After sequential selection with 240 mg/ml G418 and 2 µM gancyclovir, ES cell clones targeted correctly were identified by Southern blotting using *Eco*RV restricted ES cell genomic DNA and a pROSA-5′ probe [15]. To test the functionality of the *lox*P sites and the dual fluorescent reporter gene, we performed a transient transfection of a Cre-expressing plasmid (pOG231) into the targeted *R26R-GR* ES cells to excise the PGK-neo stop cassette [50]. The resulting ES cell clone emits dual fluorescence in the predicted pattern, i.e. nuclear localization of EGFP and plasma membrane localization of mCherry. Two independent targeted ES cell clones were used for injection into C57BL/6 blastocysts. Chimeric males were bred with C57BL/6 females to produce heterozygous *R26R-GR* mice. Germline transmission was identified using a PCR genotyping strategy. *R26R-GR/+; Sox2Cre^tg^/+* compound heterozygous mice were produced by mating *R26R-GR* males with *Sox2Cre* transgenic females [24]. The experimental protocols used in the present study were approved by the Institutional Animal Care and Use Committee (IACUC) National Taiwan University College of Medicine and College of Public Health, Taipei, Taiwan.

### PCR Genotyping

To identify the *R26R-GR* allele, a three-primer genotyping strategy was designed (Figure1 A). Primer sequences used in this study were: P1, 5′-GTT CGT GCA AGT TGA GTC CAT CC-3′; P2, 5′-CAC CAT CGT GGA ACA GTA CGA-3′; and P3, 5′-GAA GTC TTG TCC CTC CAA TTT TAC AC-3′. The wild type allele amplified by primer pair P1–P3 produces a PCR product of 622 bp whereas the *R26R-GR* allele amplified by primer pair P2–P3 gives a 1,087 bp -band. All PCR reactions were performed using the following conditions: 94°C for 1 min followed by 35 cycles of 94°C for 30 s, 60°C for 30 s, and 72°C 1 min. The results are presented in Figure 1E.

### Fluorescence Microscopy, Immunofluorescence Staining and Image Processing

E17.5 embryos were dissected and P19 mice were anaesthetized with 2.5% avertin (10 ml/kg). Whole embryos or organs including brain, intestine and thoracic vertebrae were removed and fixed in 4% paraformaldehyde in phosphate buffered saline (PBS) overnight at 4°C. The organs were cryoprotected in 25% sucrose, frozen and mounted with a Tissue-Tek OCT compound (Ted Pella. Inc., CA, USA), and cut to 5 µm thickness with a cryomicrotome. Slides were rinsed in PBS for 5 min and mounted with fluorescence mounting medium (Dako, Glostrup, Denmark). For immunofluorescence staining, mounted frozen sections were thawed and rehydrated in PBS and permeabilized with PBST (0.3% Triton X-100). After incubating the slides with blocking solution (10% normal goat serum and 2% bovine serum albumin (BSA) in PBST) for 2 h at room temperature, sections were incubated with an anti-calnexin or an anti-EEA1 antibody (both 1∶100; Genetex, San Antonio, TX, USA) in a humidified chamber for 12 h at 4°C. The sections were then incubated with Alexa 647 conjugated goat anti-rabbit IgG secondary antibody (1∶200; Molecule Probes Inc., Eugene, OR, USA). Confocal images were acquired on a Leica TCS SP5 confocal microscope, using a Leica 63X oil-immersion objective. Images were adjusted using XnView software (http://www.xnview.com).

### E10.5 Embryo Imaging

The images of E10.5 embryos were acquired by a fluorescence stereomicroscope (Leica MZ-16FA; Leica Microsystems, Wetzlar, Germany) with a Leica DFC 490 camera. The embryos were then fixed in 4% paraformaldehyde-PBS for 4 h at 4°C. The organs were cryoprotected in 30% sucrose, frozen and mounted in Tissue-Tek OCT compound (Ted Pella. Inc., Redding, CA, USA), and cut to 5 µm thickness with a cryomicrotome. Slides were rinsed in PBS for 5 min and mounted with fluorescence mounting medium (Dako, Glostrup, Denmark). Confocal images were acquired with ten 0.5 µm z sections on a Leica TCS SP5 confocal microscope, using a Leica 63X oil-immersion objective.

### Oocyte Imaging

*R26R-GR/+* mice were mated with *Zp3-Cre* transgenic mice, in which expression is driven by a developing oocyte-specific *zona pellucida 3* (*Zp3*) promoter [25]. The ovaries of offspring carrying *Zp3-Cre* and *R26R-GR* alleles were dissected and fixed in 4% paraformaldehyde-PBS for 1 hour at 4°C After cryoprotected in 30% sucrose, ovaries were frozen and cut to 20 µm thickness with a cryomicrotome. Slides were rinsed in PBS for 5 min and mounted with fluorescence mounting medium (Dako, Glostrup, Denmark). The images were acquired by a fluorescence microscopy (DMR, Leica Microsystems, Wetzlar, Germany) using a DP72 CCD (Olympus Corporation, Tokyo, Japan) and processed by Photoshop software (Adobe Systems Incorporated, San Jose, CA, USA).

### Embryo Culture and Imaging

For collection of *R26-GR/+* embryos, 3 to 4 week-old C57BL/6 female mice were induced to superovulate [51]. Zygotes were collected from the ampulla of oviducts at 0.5 dpc and cultured in KSOM medium (GIBCO/Invitrogen, Madison, WI, USA). Static imaging was acquired on a Leica TCS SP5 confocal microscope, using a Leica 63X oil-immersion objective, at 37°C and 5% CO_2_ in a humidified chamber. 488-nm and 561-nm lasers were separately used to acquire EGFP and mCherry images. For 3D image construction, the images of a blastocyst were acquired with fourteen 2.5 µm z sections and processed by Volocity software (PerkinElmer, Waltham, MA, USA).

### Mouse Embryonic Fibroblst Culture and Imaging

Homozygous *R26-GR* embryos were harvested at E13.5. Head and abdominal viscera were removed, cells were dissociated by trypsin/EDTA treatment and were cultured in DMEM with 10% FBS on a glass bottom dish (MatTek Corporation, Ashland, MA, USA). MEFs were cultured in an incubation imaging system at 37°C with 5% CO_2_. Live-cell imaging was performed with an inverted microscope AxioObserver Z1 (Carl Zeiss, Oberkochen, Germany) equipped with a 20X objective lens, an EM charged-coupled device camera (Evolve 512)(Photometrics, Tucson, AZ, USA), using 488-nm diode and 561-nm diode lasers, and triple band filter (77HE GFP/mRFP/Alexa 633 shift free). Images were acquired every 2 min with three 5 µm z sections by using AxioVision software (Carl Zeiss, Oberkochen, Germany). Images were then processed by the same software.

### Dextran Uptake Assay

Homozygous *R26-GR* mice were gavaged with 0.1 mL of 5 mM cascade blue-labeled dextran (10,000 MW; Molecule Probes Inc., Eugene, OR, USA) prepared in PBS. After 2 hours, the mice were anesthetized and then their small intestines were excised and fixed in 4% paraformaldehyde-PBS for 1 h at 4°C. The organs were cryoprotected in 25% sucrose, frozen and mounted in Tissue-Tek OCT compound, and cut to 6 µm thickness with a cryomicrotome. Slides were rinsed in PBS for 5 min and mounted with fluorescence mounting medium. Confocal images were acquired on a Leica TCS SP5 confocal microscope, using a Leica 63X oil-immersion objective. Images were adjusted using XnView software.

## Supporting Information

Figure S1 A long PCR approach to check the integrity of the 3′genomic region of the *R26R-GR* allele. The R26 targeting event was reconfirmed by a long PCR experiment using primer sequences from the report construct (P4) and a 3′external genomic region (P5) **(A)**. Only the successfully targeted *R26R-GR* allele will result in a 9.4 Kb PCR product **(B)**. Genomic DNA from the germline transmitted ES cell clone was used in this study.
(PDF)

Figure S2 Sequence analysis of an *in vivo* Cre-mediated *GR* reporter activation event. The Cre-mediated reporter activation event was examined by a PCR reaction using primer sequences anneal to the *lox*P flanking region of a *R26-GR* allele (P1 and P6) **(A)**. Only the successfully recombined *R26-GR* allele will result in a 914 bp PCR product **(B)**. The P1–P5 amplified PCR product was subject for sequence in both direction using either a P1 primer (forward) or a P6 primer (reverse). The sequencing results confirmed the successful Cre-mediated recombination between the *lox*P sites brought the *H2B-EGFP-2A-mCherry-GPI* reporter directly downstream of the *R26* promoter region and the splice acceptor sequence.
(PDF)

Figure S3 Western blotting results indicate that the 2A peptide effectively dissociates the two moieties of the dual fluorescent reporter protein. To demonstrate that the self-cleavable 2A peptide sequence allows the dissociation of the H2B-EGFP and mCherry-GPI moieties that they can appear in different subcellular localizations, antibodies against EGFP and mCherry were used to detect the resulting protein molecular weight on Western blots.
(PDF)

Figure S4 Ubiquitous dual fluorescent reporter expression in the hind limb of an E10.5 *Sox2CreERT2/+; R26R-GR/+* compound heterozygotes embryo. Note that the mCherry signal is increased in the ectoderm-mesenchyme boundary and a ventral mesenchymal mass of the hind limb bud. Scale bar: 75 µm.
(TIF)

Figure S5 Ubiquitous expression of the dual fluorescent reporter in six major organs of an adult mouse. Scale bar: B panels, 50 µm.
(TIF)

Figure S6 Distinct mCherry-GPI distributions in different *R26-GR/+* adult tissues. (A) cardiac muscle (B) back skin (C) acinar cells in salivary gland (D) intestinal epithelia (E) skeletal muscle. Scale bars: 50 µm.
(TIF)

Figure S7 H2B-EGFP images of *R26-GR* embryonic stem cells at different cell cycle stages. Live imaging of identical mES cells with nuclei expressing H2B-GFPat different cell cycle stages. The results were obtained by a Leica TSC SP5 Confocal Microscopy System equipped with a 63× oil objective. (A) Interphase (right-bottom), in which nuclear membrane was still intact, and the chromatin had not yet condensed; Prophase (left-top), in which the chromatin condensed into highly ordered structure, chromosomes; (B) Prometaphase (left-top), in which the nuclear envelop broke into fragments and disappeared. (C) Metaphase (left-top), in which condensed and highly coiled chromosomes were aligned. (D) Anaphase (left-top), in which the chromatids separated from each other and move toward the opposite ends of spindle poles.
(PDF)

Figure S8 Conditional induction of *R26R-GR* in adult tissues. (A,B,C,D) Dual fluorescent protein reporter labeled cells are found in the back skin hair follicle of a *K15CrePR/+; R26R-GR/+* adult mouse three weeks after an RU486 treatment. (E) Putative Sox2+ neural progenitors emit nuclear GFP signals were detected in the subgranular layer of the dentate gyrus one week after a tamoxifen induction in an 8-week-old *Sox2CreERT2/+; R26R-GR/+* mouse brain slide (arrows). Scale bars: 50 µm.
(TIF)

Movie S1 3D reconstruction of a *R26-GR* blastocyst. Scale bar: 100 µm.
(MP4)

Movie S2 Time-lapse imaging of MEFs isolated from an E14.5 *R26-GR* embryo.
(MP4)
