# Supplementary material for: R26R-GR: A Cre-Activable Dual Fluorescent Protein Reporter Mouse
Source: PLoS One. 2012 Sep 25;7(9):e46171. doi: 10.1371/journal.pone.0046171 (PMC3458011; doi:10.1371/journal.pone.0046171)
Supplement: Figure S1 — A long PCR approach to check the integrity of the 3′genomic region of the R26R-GR allele. The R26 targeting event was reconfirmed by a long PCR experiment using primer sequences from the report construct (P4) and a 3′external genomic region (P5) (A). Only the successfully targeted R26R-GR allele will result in a 9.4 Kb PCR product (B). Genomic DNA from the germline transmitted ES cell clone was used in this study. (PDF) [file pone.0046171.s001.pdf]

Figure S1

The R26 targeting event was reconfirmed by a long PCR experiment using primer sequences from the report construct (P4) and a 3’ external genomic region (P5) **(A)**. Only the successfully targeted R26R-GR allele will result in a 9.4 Kb PCR product **(B)**. Genomic DNA from the germline transmitted ES cell clone was used in this study.

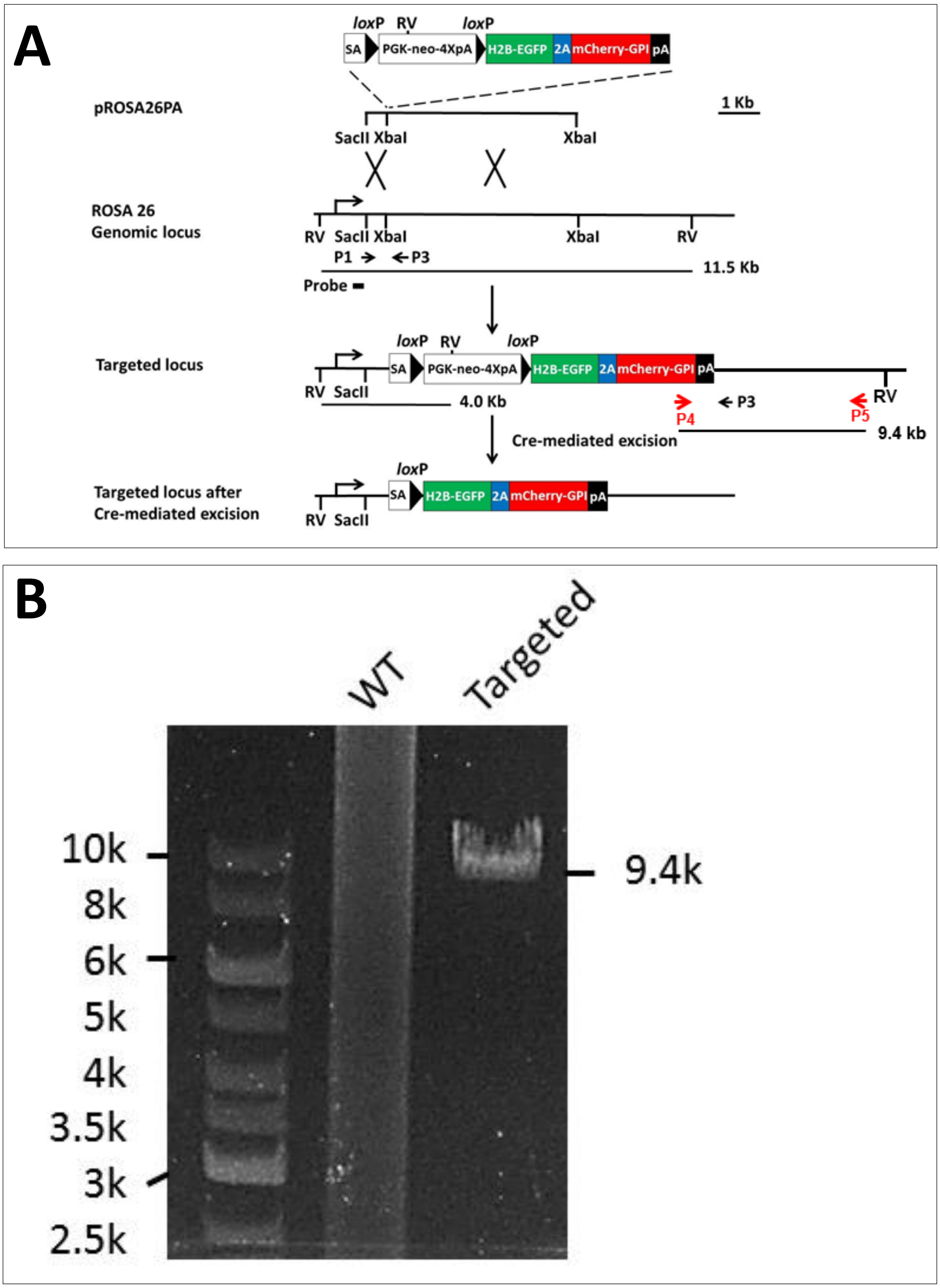

The primer sequences used in this study were the followings:

P4: 5'- CAA CGA GGA CTA CAC CAT CGT GGA ACA GTA CGA -3‘
P5: 5'- GGT TGT TTT AGA ATC AGA CTG GTA GCC TAA TGA CT -3‘

The reaction mix was the following:

Genomic DNA (dil. 10X)	5
10X Expand long template buffer	2
dNTP (10 mM)	10
$MgCl_2$(25 mM)	2
BSA (10 mg/ml)	0.5
DMSO	0.5
P4 (10 μM)	2
P5 (10 μM)	2
Expand long template enzyme mix (Roche)	1
ddH_2O	22
Total	50

The PCR reaction was performed using the following program:

92 ^oC	2’	
92 ^oC	10”	
65 ^oC	30”	10 cycles
68 ^oC	8’	
92 ^oC	10”	
65 ^oC	30”	
68 ^oC	8’+20”	25 cycles
68 ^oC	7’	
14 ^oC	∞	
