# Supplementary material for: R26R-GR: A Cre-Activable Dual Fluorescent Protein Reporter Mouse
Source: PLoS One. 2012 Sep 25;7(9):e46171. doi: 10.1371/journal.pone.0046171 (PMC3458011; doi:10.1371/journal.pone.0046171)

Figure S2

The Cre-mediated reporter activation event was examined by a PCR reaction using primer sequences anneal to the loxP flanking region of a R26-GR allele (P1 and P6) **(A)**. Only the successfully recombined R26-GR allele will result in a 914 bp PCR product **(B)**.

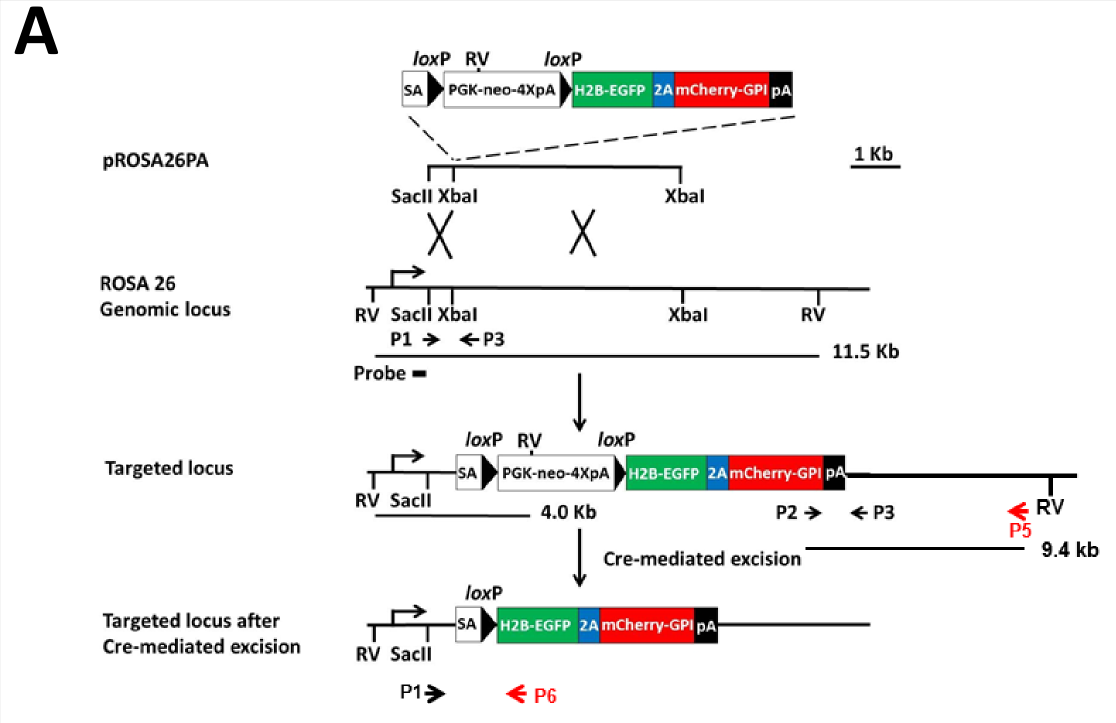

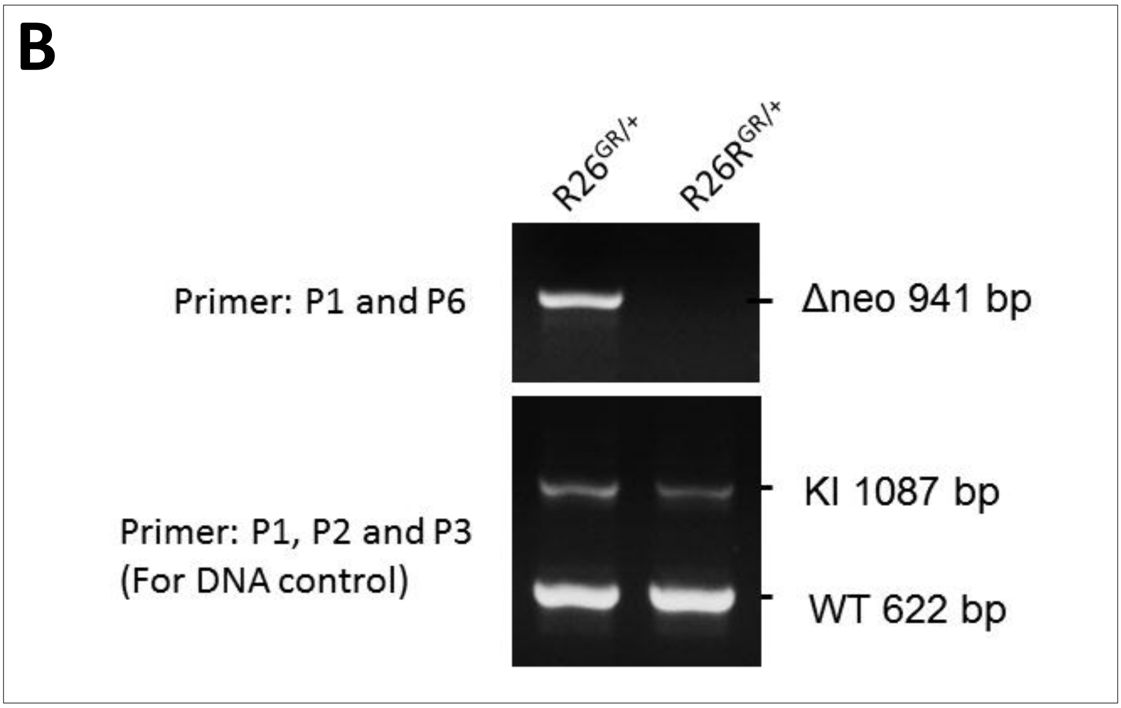

The primer sequences used in this study are the followings:

P1: 5’- GTT CGT GCA AGT TGA GTC CAT CC -3’
P2: 5'- CAC CAT CGT GGA ACA GTA CGA -3‘
P3: 5’- GAA GTC TTG TCC CTC CAA TTT TAC AC -3’
P6: 5’- ACC TGC TTC AGA ACC TTG TA -3’

The reaction mix was the following

Genomic DNA	1
10X Pfu Ultrall Fusion HS DNA Polymerase Buffer	5
dNTP (10 mM)	4
P1 (10 μM)	1
P6 (10 μM)	1
Pfu Ultrall Fusion HS DNA Polymerase (Stratagene)	1
ddH_2O	37
Total	50

The PCR reaction was performed using the following program:

95 ^{o}C	2’	
95 ^{o}C	20”	35 cycles
58 ^{o}C	30”	35 cycles
72 ^{o}C	25”	35 cycles
72 ^{o}C	3’	
14 ^{o}C	∞	

The P1-P5 amplified PCR product was subject for sequence in both direction using either a P1 primer (forward) or a P6 primer (reverse). The sequencing results confirmed the successful Cre-mediated recombination between the *lox*P sites brought the H2B-EGFP-2A-mCherry-GPI reporter directly downstream of the R26 promoter region and the splice acceptor sequence.

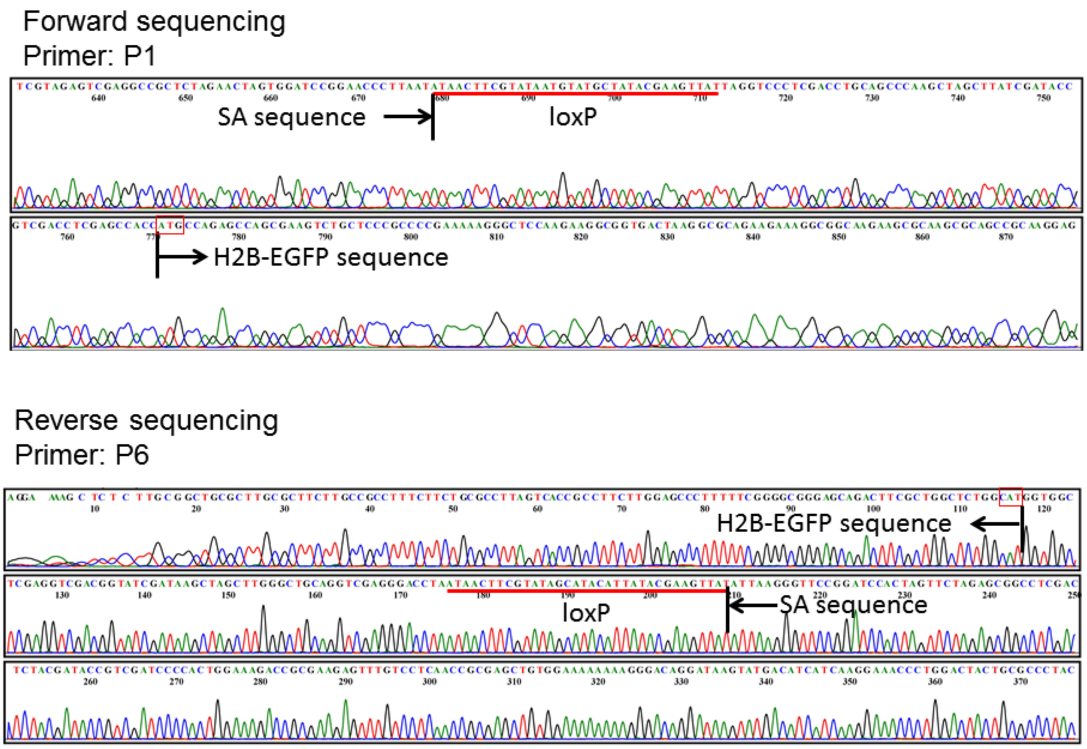

Supplement: Figure S2 — Sequence analysis of an in vivo Cre-mediated GR reporter activation event. The Cre-mediated reporter activation event was examined by a PCR reaction using primer sequences anneal to the loxP flanking region of a R26-GR allele (P1 and P6) (A). Only the successfully recombined R26-GR allele will result in a 914 bp PCR product (B). The P1–P5 amplified PCR product was subject for sequence in both direction using either a P1 primer (forward) or a P6 primer (reverse). The sequencing results confirmed the successful Cre-mediated recombination between the loxP sites brought the H2B-EGFP-2A-mCherry-GPI reporter directly downstream of the R26 promoter region and the splice acceptor sequence. (PDF) [file pone.0046171.s002.pdf]
