# Supplementary material for: R26R-GR: A Cre-Activable Dual Fluorescent Protein Reporter Mouse
Source: PLoS One. 2012 Sep 25;7(9):e46171. doi: 10.1371/journal.pone.0046171 (PMC3458011; doi:10.1371/journal.pone.0046171)

Figure S3

To demonstrate that the self-cleavable 2A peptide sequence allows the dissociation of the H2B-EGFP and mCherry-GPI moieties that they can appear in different subcellular localizations, antibodies against EGFP and mCherry were used to detect the resulting protein molecular weight on Western Blots.

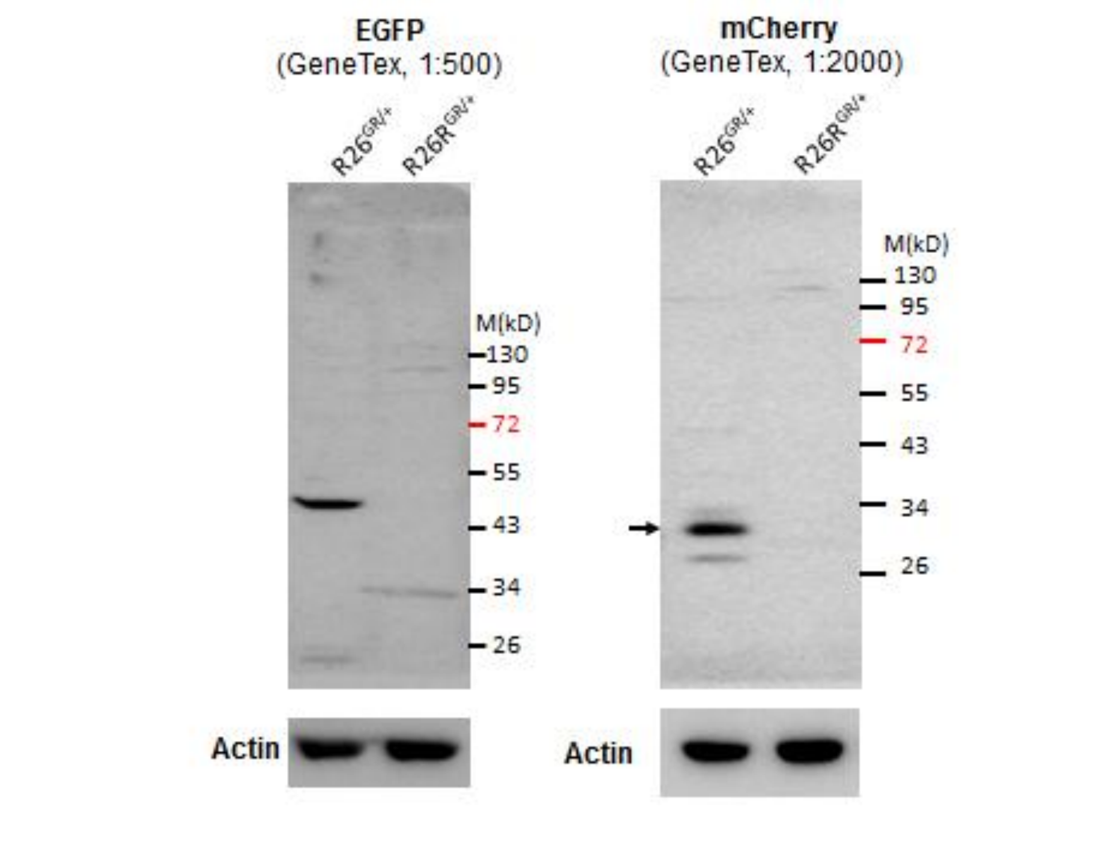

Supplement: Figure S3 — Western blotting results indicate that the 2A peptide effectively dissociates the two moieties of the dual fluorescent reporter protein. To demonstrate that the self-cleavable 2A peptide sequence allows the dissociation of the H2B-EGFP and mCherry-GPI moieties that they can appear in different subcellular localizations, antibodies against EGFP and mCherry were used to detect the resulting protein molecular weight on Western blots. (PDF) [file pone.0046171.s003.pdf]
