# Supplementary figures and images for: R26R-GR: A Cre-Activable Dual Fluorescent Protein Reporter Mouse
Source: PLoS One. 2012 Sep 25;7(9):e46171. doi: 10.1371/journal.pone.0046171 (PMC3458011; doi:10.1371/journal.pone.0046171)

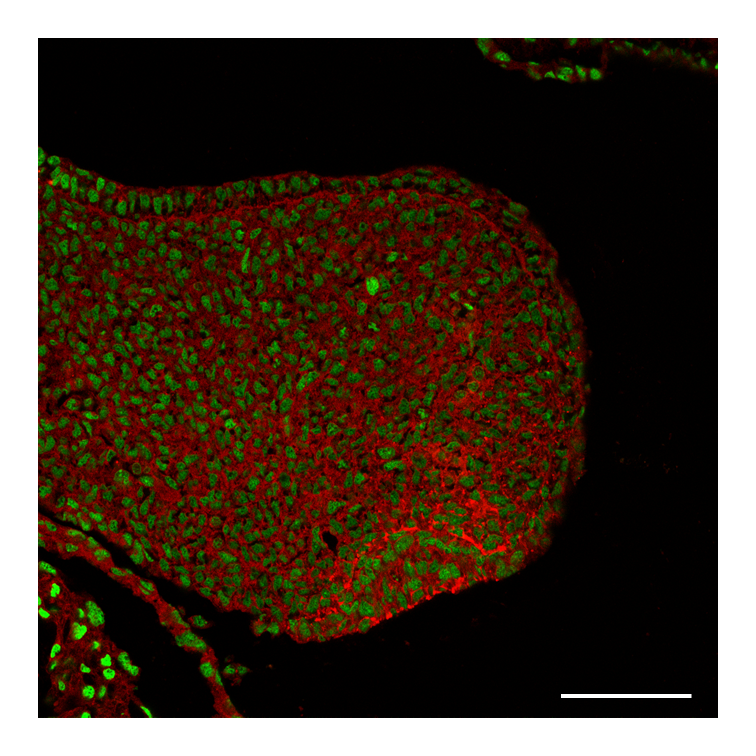

Supplement: Figure S4 — Ubiquitous dual fluorescent reporter expression in the hind limb of an E10.5 Sox2CreERT2/+; R26R-GR/+ compound heterozygotes embryo. Note that the mCherry signal is increased in the ectoderm-mesenchyme boundary and a ventral mesenchymal mass of the hind limb bud. Scale bar: 75 µm. (TIF) [file pone.0046171.s004.tif]

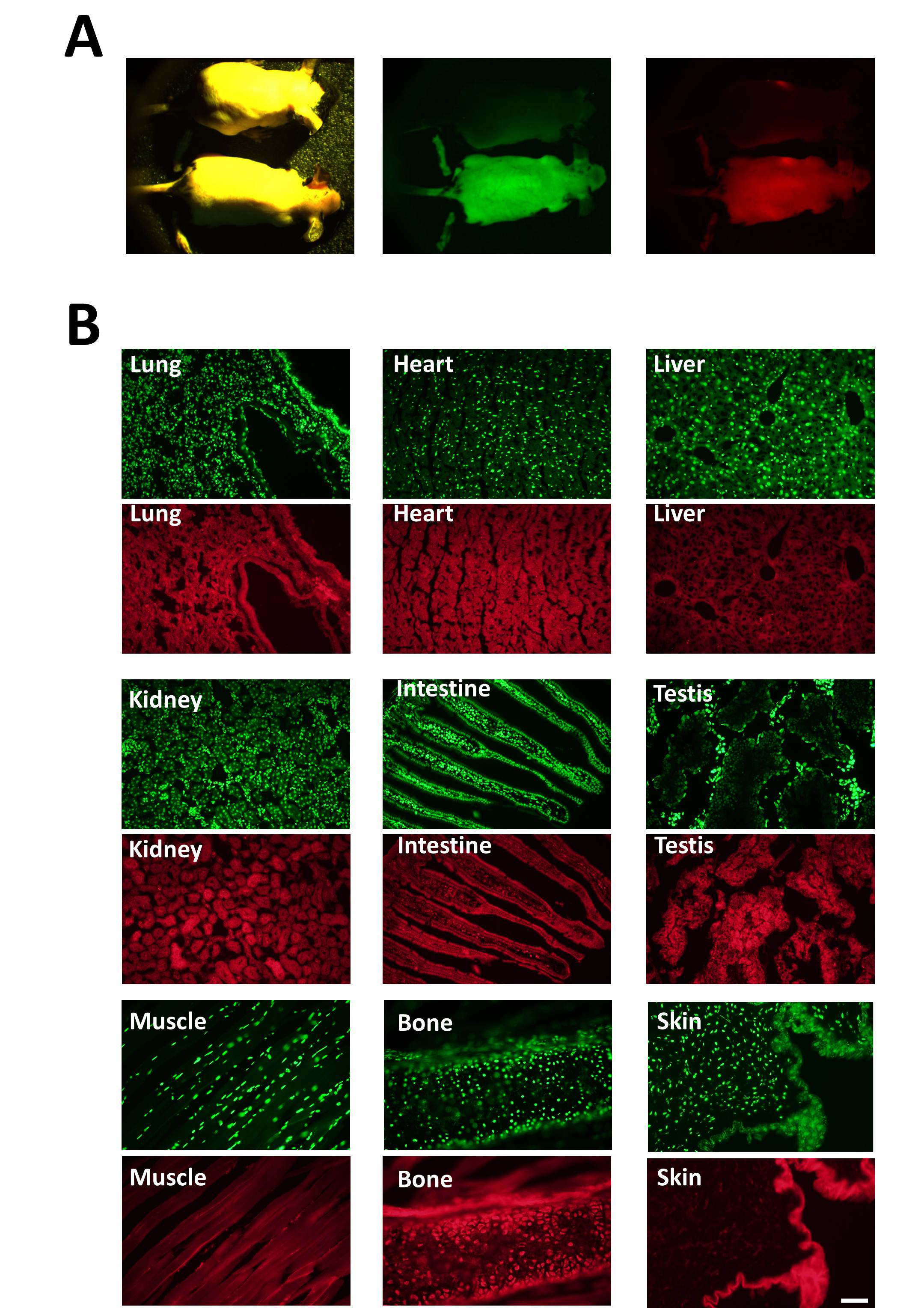

Supplement: Figure S5 — Ubiquitous expression of the dual fluorescent reporter in six major organs of an adult mouse. Scale bar: B panels, 50 µm. (TIF) [file pone.0046171.s005.tif]

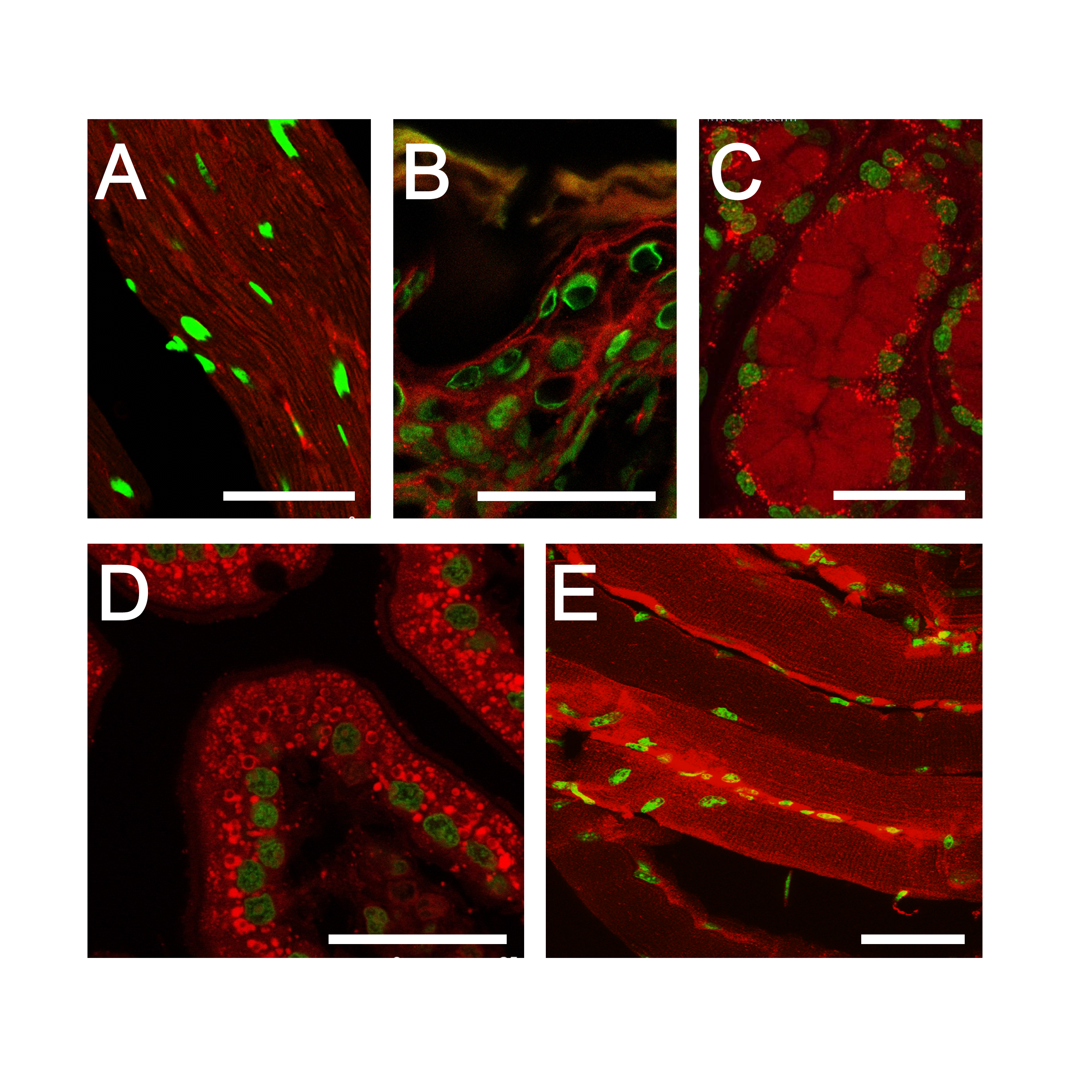

Supplement: Figure S6 — Distinct mCherry-GPI distributions in different R26-GR/+ adult tissues. (A) cardiac muscle (B) back skin (C) acinar cells in salivary gland (D) intestinal epithelia (E) skeletal muscle. Scale bars: 50 µm. (TIF) [file pone.0046171.s006.tif]

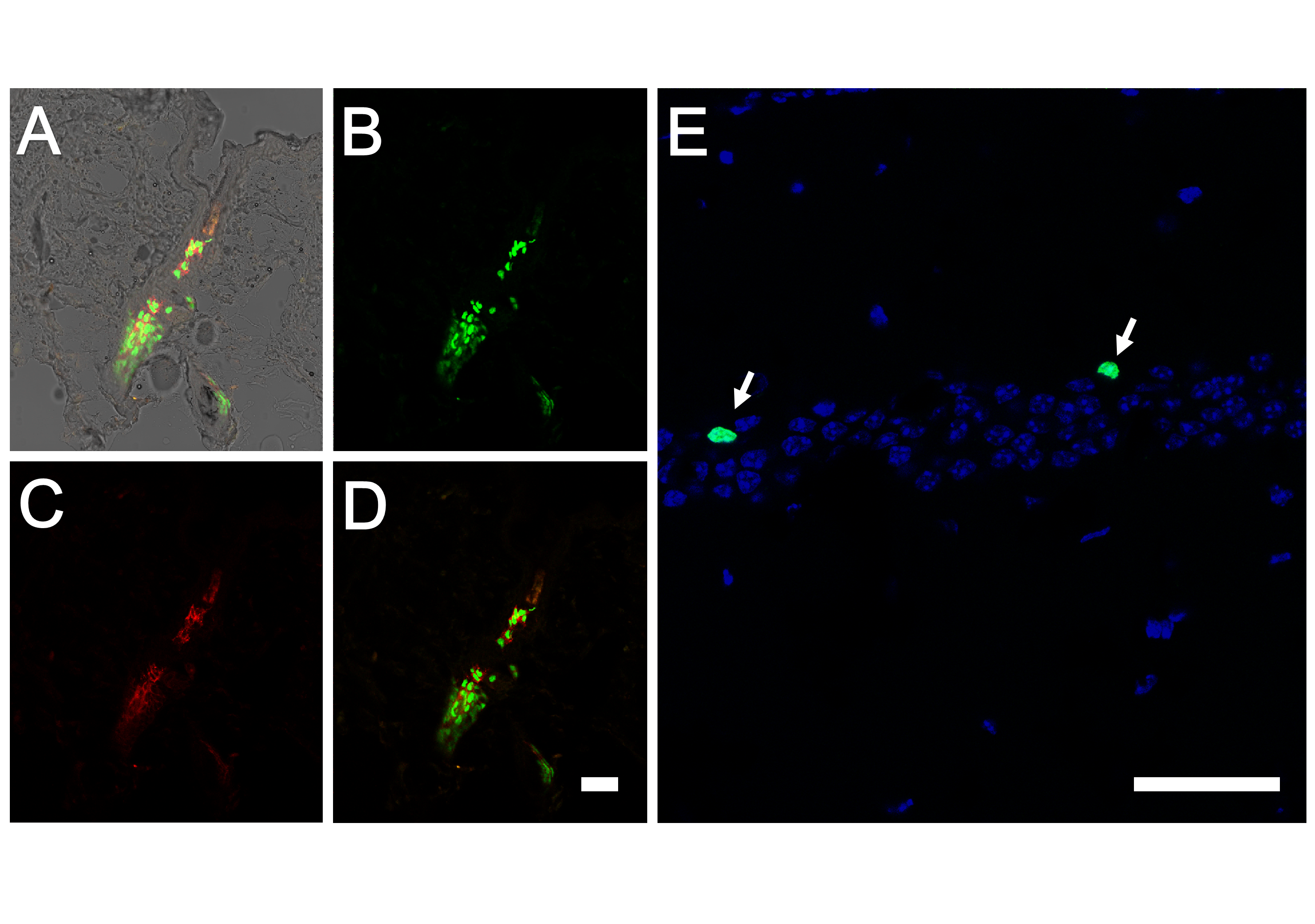

Supplement: Figure S8 — Conditional induction of R26R-GR in adult tissues. (A,B,C,D) Dual fluorescent protein reporter labeled cells are found in the back skin hair follicle of a K15CrePR/+; R26R-GR/+ adult mouse three weeks after an RU486 treatment. (E) Putative Sox2+ neural progenitors emit nuclear GFP signals were detected in the subgranular layer of the dentate gyrus one week after a tamoxifen induction in an 8-week-old Sox2CreERT2/+; R26R-GR/+ mouse brain slide (arrows). Scale bars: 50 µm. (TIF) [file pone.0046171.s008.tif]
