# Supplementary material for: R26R-GR: A Cre-Activable Dual Fluorescent Protein Reporter Mouse
Source: PLoS One. 2012 Sep 25;7(9):e46171. doi: 10.1371/journal.pone.0046171 (PMC3458011; doi:10.1371/journal.pone.0046171)
Supplement: Figure S7 — H2B-EGFP images of R26-GR embryonic stem cells at different cell cycle stages. Live imaging of identical mES cells with nuclei expressing H2B-GFPat different cell cycle stages. The results were obtained by a Leica TSC SP5 Confocal Microscopy System equipped with a 63× oil objective. (A) Interphase (right-bottom), in which nuclear membrane was still intact, and the chromatin had not yet condensed; Prophase (left-top), in which the chromatin condensed into highly ordered structure, chromosomes; (B) Prometaphase (left-top), in which the nuclear envelop broke into fragments and disappeared. (C) Metaphase (left-top), in which condensed and highly coiled chromosomes were aligned. (D) Anaphase (left-top), in which the chromatids separated from each other and move toward the opposite ends of spindle poles. (PDF) [file pone.0046171.s007.pdf]

Figure S7

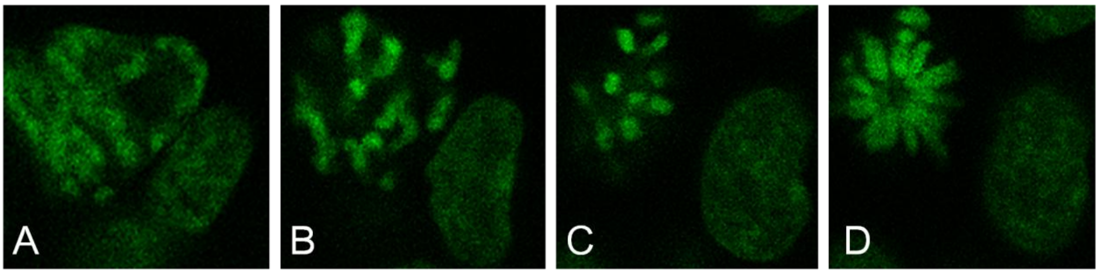

Live imaging of identical mES cells with nuclei expressing H2B-GFP at different cell cycle stages.

The results were obtained by a Leica TSC SP5 Confocal Microscopy System equipped with a 63× oil objective. (A) Interphase (right-bottom), in which nuclear membrane was still intact, and the chromatin had not yet condensed; Prophase (left-top), in which the chromatin condensed into highly ordered structure, chromosomes; (B) Prometaphase (left-top), in which the nuclear envelop broke into fragments and disappeared. (C) Metaphase (left-top), in which condensed & highly coiled chromosomes were aligned. (D) Anaphase (left-top), in which the chromatids separated from each other and move toward the opposite ends of spindle poles.
